# Social Investment Policies and Childbearing Across 20 Countries: Longitudinal and Micro-Level Analyses

**DOI:** 10.1007/s10680-022-09626-3

**Published:** 2022-06-30

**Authors:** Sunnee Billingsley, Gerda Neyer, Katharina Wesolowski

**Affiliations:** 1 Department of Sociology, Demography Unit, Stockholm University, Stockholm, Sweden; 2 Department of Sociology, Södertörn University, Huddinge, Sweden; 3 School of Humanities, Education and Social Sciences, Örebro University, Örebro, Sweden

**Keywords:** Fertility, Family policy, Social investment-oriented support, Family benefits, Fixed effects linear probability models

## Abstract

This study analyses the influence of family policies on women’s first and second births in 20 countries over the period 1995 to 2007. Welfare states have shifted towards social investment policies, yet family policy–fertility research has not explicitly considered this development. We distinguish between social investment-oriented and passive support that families may receive upon the birth of a child and consider changes in policies over time. These indicators are merged with fertility histories provided by harmonized individual-level data, and we use time-conditioned, fixed effects linear probability models. We find higher social investment-oriented support to be correlated with increased first birth probabilities, in contrast to passive family support. First birth probabilities particularly declined with higher passive family support for women over age 30, which points to a potential increase in childlessness. Social investment-oriented support is positively related to first and second births particularly for lower-educated women and has no relationship to childbirth for highly educated women, countering the Matthew-effect assumptions about social investment policies. Passive support is negatively related to second births for post-secondary educated women and those who are studying. Family policies that support women’s employment and labour market attachment are positively linked to family expansion and these policies minimize educational differences in childbearing.

**Electronic supplementary material:**

The online version of this article (10.1007/s10680-022-09626-3) contains supplementary material, which is available to authorized users.

## Introduction

The political and demographic debate on fertility developments in post-industrial societies has moved family policies to the forefront as an essential means of shaping fertility behaviour. Family policies that facilitate a gender-equal reconciliation of work and care are argued to encourage childbearing, whereas family policies that support a gendered division of work and care hamper it (Brewster & Rindfuss, 2000; Esping-Andersen & Billari, 2015; Goldscheider et al., 2015; McDonald, 2000, 2013; Neyer, 2003, 2005; Rovny, 2011). This view deviates from the perspective that centres on the economic costs of and the expenditure on children (Becker, 1981; Ermisch, 2003; Hotz et al., 1997). The shift towards a gender-egalitarian work-life perspective in family policy and fertility research coincided with shifts in welfare state research towards social-investment welfare states. These are welfare states that prioritize an employment-centred life course for men and women alike over passive social protection during times of need. Their social policies back or mandate a gender-egalitarian behaviour in all aspects of life (Esping-Andersen, 2002; Jenson, 2009; Morel et al., 2012; Saraceno, 2017). Even though welfare states are more and more moving towards social investment policies, family policy–fertility research has not explicitly incorporated this development. In line with the prevailing theoretical assumption in fertility research, one may conclude that family policies that embrace a social-investment orientation may be conducive to childbearing, whereas passive family policies that provide support without an employment and gender-egalitarian perspective may not.

Empirically, this is not easy to test; the family policies of a country are rarely either completely social investment-oriented or completely passive. In general, the support that countries offer families is a mixture of policies with different orientations. Previous research on the impact of family policies on fertility has rarely empirically modelled these simultaneously different orientations of the family policies of a country. The core interest was usually on specific policies, such as childcare, parental and care leave, and cash benefits (see Duvander et al., 2010; Gauthier & Hatzius, 1997; Luci-Greulich & Thévenon, 2013; Rindfuss et al., 2010). If one compares the results of the studies, it is often difficult to draw a definite conclusion as to the effect of these policies on fertility (see Gauthier, 2007; Sleebos, 2003). This may be precisely because within and across countries family policies may be incongruous and even similar policies may have a different orientation or a different function with respect to, e.g. parental employment, gender distribution of care, or equality of work-life balance for all (Korpi, 2000; Sainsbury, 1996; Saraceno, 2011; Wesolowski & Ferrarini, 2015). Some family policies may be more social investment-oriented, such as earnings-related parental leave, others may be more passive or directed at mothers’ retreat from employment, such as (flat-rate) care leaves, cash benefits or tax reductions for single earners. If one lumps together policies with opposing orientation, such as earnings-related parental leave and flat-rate care leave, results may be ambivalent or cancel themselves out. A similar effect may occur if one studies a single policy in a country with ambivalent family policies. To consider the degree to which a country’s family policies are social investment-oriented and to which they are passive is thus a necessary pre-condition for assessing the link between family policies and fertility.

Another issue concerns the approach and design of family policy–fertility research. Studies of the associations between family policies and fertility remain often at the aggregate level and are cross sectional (e.g. Castles, 2003; Luci-Greulich & Thévenon, 2013; Rovny, 2011). The same applies to studies on the impact of social-investment welfare states on families (see, e.g. Plavgo & Hemerijck, 2021). Researchers, even those using aggregate data in a cross-sectional design, commonly acknowledge that to assess whether family policies influence fertility, we need to investigate their impact on individual behaviour. Since the balance between social investment-oriented and passive family policies can change over time, we need to use this variation within a national context to explore their influence on childbearing behaviour in a longitudinal micro-level perspective (see, e.g. Hoem, 2008; Neyer & Andersson, 2008; Parolin & Van Lancker, 2021). To the best of our knowledge, this has neither been done in fertility nor in social-investment research and we therefore lack evidence for how shifts in policy orientations may support childbearing and for whom.

The social investment approach provides a theoretical frame to conjoin the demographic theories of employment-care reconciliation and economic support and allows us to distinguish between the different objectives that family policies may pursue with respect to childbearing. In line with this approach, we differentiate between “social investment-oriented” family policies (that are employment centred), and “passive support” policies (that ease the costs of children but have no employment focus). Our indicators measure how prevalent each of these aspects is within the family-policy configuration of a country and whether and to what extent the magnitude of each changes over time.

Our policy data come from the Social Policy Indicator Database (SPIN, 2019) and provide measures of social investment-oriented and passive support as they relate to family policies in the years 1995, 2000, and 2005. We use individual-level fertility histories drawn from the Harmonized Histories of the Generations and Gender Surveys (GGS) and Changing Life Course Regimes (CLiCR); data that cover the years from 1995 to 2007, allowing us to assess policy variation and individual fertility histories over time within 20 countries. The years comprise the period when persistently low fertility levels despite economic stability in many post-industrial countries triggered much debate and concern about suitable policy intervention to enable women and men to have the number of children they desire (OECD, 2011). The time-span also includes the turn to social investment welfare states (Morel et al., 2012) and the beginning of widespread increases in fertility rates, thus broadening variation in fertility behaviour and allowing us to contribute to the discussion on which family policies may play a role in the recovery of fertility rates (Bongaarts & Sobotka, 2012; Goldstein et al., 2009; Myrskylä et al., 2013)**.**

We use fixed effects linear probability models to trace how the set-up of family policies at one time-point influences women´s childbearing in the current and subsequent two years, controlling for unobserved heterogeneity at the country and year level as well as for individual-level factors. Since policies may work differently for first and subsequent births, for younger and older women, and for different educational groups, we explore these differences.

## The Link Between Family Policies and Fertility

Most comparative studies of family policies and fertility either explore the link between family policies and fertility at the macro-level (using the total fertility rate (TFR)) or they investigate the impact of a specific family policy on childbearing behaviour at the micro-level such as the use of child care, the level of child benefit, or the uptake of parental leave.^1^ Macro-level studies often find some positive association between family policy and TFR, although the results vary by the type of family policy, the period or the countries studied (Castles, 2003; Gauthier & Hatzius, 1997; Luci-Greulich & Thévenon, 2013; Rovny, 2011). Acknowledging the weakness of the TFR as an indicator of fertility behaviour (Hoem, 2008; Neyer & Andersson, 2008). some studies investigate the link between different types and/or generosity levels of family policy support and first or second birth rates (Adsera, 2011; Begall & Mills, 2011; Kalwij, 2010). These researchers find that different policies may be differently related to first or subsequent births.

As noted above, the lack of consistent results may be due to ignoring that the policies of a country may not always work in the same direction and that similar policies may have different objectives in different countries. For example, an earnings-related parental leave may be an employment incentive for women (and a care incentive for men), whereas the taxation policy may support gendered employment and care behaviour (e.g. in the case of Germany). Several researchers have therefore grouped family policies along different dimensions, e.g. familialisation – de-familialisation, or developed indexes to capture such ambiguities, often for cross-sectional comparisons of welfare state orientations (for an overview of such measurements, see Lohmann & Zagel, 2016). Korpi (2000) and Ferrarini (2003) suggest classifying family policies by weighing the extent to which they support a gender-equal employment-oriented life course (and thus facilitate a more gender-equal division of employment and care), vs. the extent to which they support a traditional gender division of labour. This resembles de-familialisation – familialisation approaches (Lohmann & Zagel, 2016). In addition, they point out that both of these dimensions must be represented simultaneously to avoid neglecting a potentially ambiguous constellation or over-emphasizing either orientation.

To our knowledge, only a few studies have applied the approach suggested by Korpi (2000) and Ferrarini (2003) to fertility and family research. The few studies that use their classification in the intended way put the theoretical emphasis on the different family models supported by different family policies. Wesolowski and Ferrarini (2015) find that a stronger support of the earner–carer family model is associated with higher TFR; Ferrarini (2003) shows that both high support for earner-carer family orientation as well as for the traditional family orientation is correlated with higher TFR. Billingsley and Ferrarini (2014) outline different mechanisms that link family policy orientations and fertility decision-making. They demonstrate that both types of family policy support are positively linked to individuals’ intentions to have a first child, but only earner–carer policy support strengthens individuals’ intentions to have a second child.

Following these lines of research, we employ the classification of family policies used by Korpi (2000), Ferrarini (2003), Wesolowski and Ferrarini (2015), and Billingsley and Ferrarini (2014) and incorporate it into the broader theoretical framework of social-investment welfare policies. The major distinction that we derive from the social-investment approach is between family policies that take an employment orientation and those that do not. In most countries, the gender aspect in social-investment policies is still not fully supporting a gender-egalitarian earner–carer behaviour, which lends a conceptualization related to employment support more accurate than one related to gender egalitarianism. The social investment approach is therefore a significant contribution to the theoretical development in family and fertility research, bridging new welfare state theories and social-demographic theories. It offers a unique interdisciplinary approach to incorporate the new and emerging logics of family policies in post-industrial welfare states into family and fertility research.

## Theoretical Considerations and Expectations

The social investment approach unites and extends the economic, demographic, and gender-related aspects of family policy and fertility research. The origin of this approach can be traced back to the fertility crisis of the 1930s and Alva and Gunnar Myrdal’s suggestion to view social policies not as a cost factor but as an investment in the productivity of the population (Morel et al., 2012). It revived in the 1980s and early 2000s, when feminist and welfare state researchers called for changes in the welfare state from passive protection towards policies that actively promote women’s employment, increase gender equality, reduce social inequality, and centre more on fertility and children to sustain the welfare state (see, e.g. Esping-Andersen, 2002; Hernes, 1987).

Passive social policies have been primarily providing financial support in case of “old” social risks, e.g. loss of income due to unemployment or illness. Family policies in passive protection-oriented welfare systems are largely based on the notion of the male breadwinner – female carer family (Jenson, 2009). They tend towards supplementing the income of the family to reduce the costs of having and caring for children. These policies typically favour flat-rate cash transfers of various types, like birth grants, child allowance, care grants, marriage subsidies or tax deductions for the main earner. In general, passive family policies are unrelated or only weakly related to employment and previous income and do not explicitly support mothers’ labour-force participation or facilitate fathers’ care engagement (Ferrarini, 2003; Korpi, 2000).

Social investment-oriented policies focus on life-long employment for women and men alike. They aim to enable them to be employed throughout their employable lifetime, irrespective of their family obligations (Beramendi et al., 2015; Morel et al., 2012). Their main objective is thus not to passively compensate for income loss, but to target the social risks that may impede a person’s labour-force participation over her life course. Entering parenthood and having a child to raise are regarded as such a social risk (Bonoli, 2005). Social investment-oriented family policies regard each parent from an employment perspective (Bonoli, 2005; Morgan, 2012), and as such, they take an inherently gender-egalitarian orientation (Jenson, 2009; Saraceno, 2017). Their goal is to enable women (and men) to have and care for a child without cutting their ties to the labour market, compromising their long-term employability and economic security (Bonoli, 2005; Jenson, 2009, 2012; Morgan, 2012; Saraceno, 2017).

The employment focus of social investment policies has raised controversy among social scientists. Some researchers find that social-investment welfare states reduce gender and social inequality and level out class differences (Korpi et al., 2013; Plavgo & Hemerijck, 2021). Others argue that the social investment strategy imposes an adult male worker model on everyone, exerts pressure on women and mothers to be employed, devalues and sidelines familial care, and lacks an encompassing gender equality agenda (Daly, 2011; Jenson, 2009; Saraceno, 2015). Some point to potential Matthew effects, that is, that social investment policies disproportionately benefit middle-class and better-off families and disadvantage those who are more dependent on passive support, such as the low educated (Bonoli et al., 2017; Cantillon & Van Lancker, 2013). These propositions have been mainly derived from aggregate and often cross-sectional research. Micro-level and longitudinal investigations necessary to substantiate these claims are still lacking (on the need for such research, see Parolin & Van Lancker, 2021).

Researchers of social investment welfare states have primarily looked at the role of public childcare as a tool to promote mothers’ employment (see Hemerijck, 2017). However, studies have shown that the birth of a child is the point of departure in women’s lives that determines their future employment trajectories. Family policies connected to the birth of a child have turned out to be important means to shape employment and gender behaviour after childbearing (e.g. Aisenbrey et al., 2009; Budig et al., 2012; Hook, 2006, 2010). Social investment policies around the birth of a child usually comprise maternity leave and increasingly also parental leave, both with benefits based on one’s previous income. The benefits are usually considerably higher than the flat-rate benefits of passive family policies, not least because they aim to incentivize women/mothers to take up employment prior to having a child and return to the labour market thereafter, to provide income security despite birth-related absence from paid work, and to encourage fathers to take parental leave. These perspectives are largely absent in passive family policies based on flat-rate benefits (see, e.g. Budig et al., 2012; Morgan, 2012; Morgan & Zippel, 2003; Ziefle & Gangl, 2014).

Post-industrial welfare states nowadays usually employ passive family policies, such as child benefits or tax reductions for families with children, in addition to employment-related policies, such as maternity leave. Welfare state researchers generally have noted a shift of welfare states towards strengthening social investment aspects of their welfare policies (see, e.g. Morel et al., 2012). The transformation towards social-investment welfare states, however, has neither been linear nor uniform across post-industrial countries (Morgan, 2012). Changes were often incremental and/or only concerned specific parts of the policies (e.g. introduction of paternity leave alongside a traditional flat-rate parental leave). For example, the Nordic countries started to implement pronounced social investment-oriented family policies as early as the 1970s. In Eastern Europe, the socialist variants of employment-focussed family policies were largely abandoned after the fall of state socialism. Subsequent changes between these policy orientations have been rather frequent in some countries, so that they have oscillated between being more familistic-care or more social-investment promoting (Rostgaard, 2004; Szelewa & Polakowski, 2008). Continental Western and Southern European countries have mostly been “slow movers” in the time-span of this study (1995–2005) (Morgan, 2012) towards some social investment components in their systems.

Taking these considerations into account, we formulate expectations related to the potential relationship between childbearing and passive vs. social investment-oriented family policies. First, demographers argue that with women’s increasing education and labour-force participation, passive family support may hamper childbearing because these policies do not facilitate women’s employment retention and may enlarge gender inequality (Goldscheider et al., 2015). Social investment-oriented policies should work in the opposite direction, so that childbearing rates should be higher when countries have implemented social investment-oriented policies.

Since the financial benefits to (new) parents in social investment-oriented welfare states are usually income related, and women without children commonly work (after having attained their education), this may particularly apply to first births. We may, however, expect women living in such contexts to postpone having their first child until they are established in the labour market and/or have a sufficiently high income. In other words, we expect a higher propensity to have a first child at higher ages in countries with high social investment support. The prevalence of passive family policies may be an incentive to have one’s children before one is firmly established in the labour market, so that employment is not interrupted for a long time and income losses are minimized. The timing of parenthood may have important implications for women’s career and wage developments since research has shown women who delay first births are likely to have higher earnings after becoming a mother than women who do not (Amuedo-Dorantes & Kimmel, 2005; Miller, 2011).

Furthermore, considering the Matthew effect argument, one may expect first birth differences between low and highly educated women in countries with strong social investment-oriented family policies, because better-educated women are assumed to benefit more from an earnings-related parental leave than low-educated women. Since social investment policies aim at maintaining the caring parent’s income during leave, reducing economic uncertainty, retaining employment and career potential beyond the childbearing period, and promoting gender-equal life-courses of employment and care (Korpi et al., 2013). one may also expect no first birth differences between low and highly educated women in countries with strong social investment-oriented family policies.

Since many women reduce their employment after becoming mothers, the relationship between the type of family support and second birth propensity may be less clear-cut than for first birth. Nevertheless, applying the reasoning that social-investment policies provide employment and income stability, promote gender equality, and level out social inequality (Korpi et al., 2013). we may assume that second birth rates are higher (over time or across countries) when stronger social investment-oriented policies are adopted rather than stronger passive family policies and that there should also be no differences in second-birth rates among different educational groups. If one follows the Matthew effect reasoning, one may assume a higher second-birth propensity of highly educated than of low educated women in countries with more social investment support. If passive family support prevails, we would expect second birth rates to be particularly lower among the highly educated because of the potentially more negative long-term career consequences if they intend to refrain from employment twice due to care.

## Data and Method

### Family Policy Dimensions

We investigate the time period 1995 to 2007, which is when we have comparable data available. The data on family policies for the years 1995, 2000, and 2005 are taken from the Social Policy Indicator Database (SPIN, 2019). which was developed at Stockholm University, complemented by data for Eastern Europe. The data provide two measures of financial incentives for families, both are related to the birth of a child: one represents social investment-oriented support, the other passive family support.

*Social investment-oriented support* includes earnings-related family policy transfers, namely maternity leave and parental leave benefits. We excluded paternity leave — that is days that the father is entitled to around the time of the birth of the child to either assist the mother or to take care of other children during the first weeks after the delivery — because these days are usually granted simultaneously with maternity/parental leave. Leave that is reserved for either parent as a non-transferable individual right, also called the father´s quota, is included in parental leave. Maternity leave and parental leave benefits are employment-related, and the amount a parent receives depends on her/his pre-leave income. The paid leave period differs in length from country to country and may also vary within a country over time. Because both maternity and parental leave are commonly job protected and the transfers are related to previous earnings, they also provide incentives for women to enter and stay in the labour market before becoming a mother (Bäckman & Ferrarini, 2010).

Social investment-oriented support is calculated as the sum of the annual net amounts of earnings-related post-natal maternity and parental leave benefits paid during the first year after childbirth. The net replacement rate thus considers the duration of the benefit during the first year after childbirth. A shorter duration results in a lower replacement rate even if the formal replacement rate for one week is 100% (i.e., 100% for 6 weeks results in a lower replacement rate than 90% for 20 weeks when divided by an annual wage). High replacement rates are not commonly found for periods of leave much longer than a year, and we do not consider leave payments in the second or third year in the measure. Longer leave lengths may not align with the principles of social investment-oriented support as there is some evidence that labour market attachment can be negatively affected (Nieuwenhuis et al., 2017; Pettit & Hook, 2005; Thévenon & Solaz, 2013). To capture the full degree of earnings-relatedness, the parent on leave is assumed to have worked two years before childbirth, earning an average production worker´s wage, before spending a leave period with the new born.

As an independent variable in our analyses, social investment-oriented support represents the net replacement rate of these family policies after tax, measured as a percentage of an average production worker’s net wage. The benefits are calculated according to the rules stated in the legislation of the country for a model family with two adults, of whom one is working full-time and one is on leave, and two children, of which one is new born and the other one is below school age. Taking taxation into account avoids mixing taxable and non-taxable benefits, which otherwise would bias the comparison of the benefits (Ferrarini et al., 2013). Moreover, by calculating net replacement rates for a model family these indicators avoid using replacement rates that might not be applicable to a typical wage earner with an income above the earnings ceiling (Wesolowski & Ferrarini, 2015).

In the welfare state literature, high-quality public childcare is also considered part of the social-investment-oriented family policies. Data on childcare coverage for children below the age of two are, however, unavailable widely, and childcare for children below age two is essential for re-entry into the labour market after birth, since longer absence has severe consequences for future employment and income trajectories (e.g. Aisenbrey et al., 2009). Childcare is therefore excluded from our family policy measures.

*Passive family support* comprises family policy transfers that are not related to previous employment. These include child care leave allowances (also called home care allowances or cash-for-care allowances), which are usually paid in low flat-rate amounts. These leaves may be offered after earnings-related maternity and/or parental leave; but they may also be granted irrespective of any previous employment (see also Morgan & Zippel, 2003). Due to their (low) flat-rate benefit, these leaves are usually taken by mothers, even if fathers have a right to take them as well. We also consider lump-sum grants that are paid in connection with childbirth as a passive family policy. We include cash and fiscal child benefits and tax deductions for a main earner with an economically inactive or less active partner or spouse within our category of passive family policies. These latter benefits are often referred to as “marriage subsidies” and are argued to promote female homemaking as they privilege family forms with a (usually married or legally acknowledged heterosexual cohabiting) earner and a less economically active spouse or partner. We furthermore allocate cash and fiscal child benefits to passive family policies. They aim to reduce the costs of children, but they may also be a disincentive to continue working after having a child, in particular, if the allowance is high (compared to a woman’s average income or to costs of childcare) or increases with the number of children.

The net replacement rates of lump-sum grants paid in connection with childbirth, marriage subsidies and cash and fiscal child benefits are calculated for the first year after childbirth, as in the case of social investment-oriented support. However, the flat-rate benefit for a childcare leave takes into account how much of an average net wage is replaced during the first year after the termination of earnings-related parental leave since it usually cannot be taken simultaneously with an earnings-related parental leave benefit. The net replacement rate of passive family support is thus the sum of those separate replacement rates.

The operationalization of passive policy support follows the same logic as the net replacement rate of an average production worker for a model family. For a detailed discussion of the advantages and the shortcomings of the calculation of the benefits and of using net replacement rates of a typical wage-earner’s income and for a model family, see Wesolowski et al. (2020); for a general discussion of issues related to comparative net replacement rates based on average workers’ earnings, see Ferrarini et al. (2013). The two independent variables representing the policies are centred on their respective means in the analyses.

Supplementary Figure A displays the measures in the three periods we include in our analyses, 1995, 2000, and 2005 for the 20 countries included. Both radical increases and declines in both dimensions of support appeared over our time frame. A more thorough overview of how these data represent individual policies over time within selected countries can be found in Wesolowski et al. (2020).

### Individual-Level Data on Fertility Histories

We match these policy data to women’s childbearing histories, which are derived from two harmonized data sets that were constructed using the same procedures: the Harmonized Histories (Perelli-Harris et al., 2011 and see www.nonmarital.org) and the Changing Life Course Regimes in Eastern Europe (CLiCR) (Duntava & Billingsley, 2013). Both sources rely on publicly available survey data that include questions about women’s childbearing histories from age 16 onwards. The main source for Harmonized Histories used in this study are the Generations and Gender Surveys, but other sources were used as well including the Spanish Fertility Survey, The US National Survey of Family Growth, German Pairfam, and the British Household Panel Survey. From the Harmonized Histories, we use data on Austria, Belgium, France, Germany, Norway, Spain, Sweden, UK, and US. The original sources of CLiCR data are the Family and Fertility Surveys, Generations and Gender Surveys, and Life in Transition Surveys. From CLiCR, we have data on Bulgaria, Czech Republic, Estonia, Hungary, Latvia, Lithuania, Poland, Romania, Russia, Slovenia, and Ukraine. We selected all countries that are represented in both SPIN data over time and in either the Harmonized Histories or CLiCR data.

All women for whom we can observe fertility histories (no missing information on birth dates) from the age of 16 are analysed. For the analysis of the transition to parenthood, the sample includes 50,178 women, of which 28% entered parenthood during the observation period. The birth cohorts included are from 1950 to 1993. For the second birth transition, the sample includes 33,853 women who were observed after having a first child, of which 30% conceived a second child during the observation period.

### Method

We observe all women from the month they turn 16 until they were interviewed, turn 45, or nine months before their first child or second child was born to reflect conditions at the time of conception. A discrete-time event history analysis approach is applied in which each observation represents a person/month and the value of variables can change on a monthly basis. These individual-level person/months are nested within country/year observations. We do not nest months within persons because the outcome is consistently zero until the event occurs and the person is censored, as is the case in an event history analysis set up. To reduce the influence of confounding factors and unobserved heterogeneity across countries, we use fixed effects at the country-level. Due to splitting observations into time periods, the event of interest becomes relatively rare. Because of difficulties estimating logistic fixed effects models on data comprised of rare events, we use fixed effects linear probability models (LPM). Neither fixed effects event history modelling nor LPMs are common in demographic research. Partly this is precisely because the outcome measured is a rare event, but also because LPMs can give predictions that are outside the range of 0 to 1, which is not possible in reality. We believe this shortcoming is outweighed by the possibility to remove the vast unobserved heterogeneity we are dealing with when modelling countries by using LPM with fixed effects, as well as to compare across models (Mood, 2010).

This modelling approach allows the relationship between policy measures and conceptions leading to births to be estimated only on the basis of deviation over time from the country-specific mean of the policy value. Differences between countries therefore do not influence the estimate of the relationship, as results represent an average that is net of contextual differences. In other words, fixed effects modelling adjusts for time-constant unobserved factors such as societal norms, values, institutions as well as the labour market setting at the national level. This approach also accounts for expected differences in the post-socialist countries, including the wide range in the pace of how the postponement of parenthood advanced after the collapse of the Socialist regimes (Billingsley, 2010; Billingsley & Duntava, 2017; Frejka, 2008). Fixed effects for years are included as well to independently account for shared period effects across countries such as widespread economic crises and secular changes in fertility.

We restrict the analysis to specific years in order to limit the possibility of unmeasured policy changes; policies are measured in SPIN once every five years (1995, 2000 and 2005). We therefore assess women’s first or second conception propensity from 1995–1997, 2000–2002 and 2005–2007. Each woman can contribute to different sets of years depending on her parity; a woman who is childless in 1995 will be observed until 1997 and observed again from 2000–2002 and 2005–2007 if she remains childless. If she enters parenthood in 2001, she will contribute to the second parity transition model (the transition from first to second child) for the months after the first birth in 2001 as well as in the years 2002 and 2005–2007 unless censored. This data set-up creates left-truncation of observations when a woman has a childbearing event before the years we observe. This naturally entails some loss of women who had a birth before these periods, potentially biasing our sample towards missing births at younger ages. But given that the sample selection is intermittent and repeated (all those at risk for three consecutive years selected after a two year break), we have repeated opportunity to capture women of all ages who are at risk of childbirth.

Age of the respondent is categorized into five-year age groups and used as the baseline probability for first conceptions. In the analyses of second birth conceptions, we instead use years since the first child was born and control for age at first birth.

We include dummies to capture whether or not a respondent was still studying and, if not, her educational attainment: “secondary or lower” includes less than or completed secondary school; “post-secondary” includes those who attended a higher educational institution for less than three years; “higher education” includes those who attended at least 3 or more years of university. We adjust for potential non-proportionality of the effects of educational level across the age distribution by interacting education and age (or age at first birth) in all models. Descriptive statistics of all variables are presented in Supplement A.

## Results

We first briefly discuss the average associations between the family policy measures and parity transitions (Table 1). Full model results are displayed in Supplement B and C. Social investment-oriented support is positively related to first birth conceptions, whereas passive family support is negatively related to first birth conceptions. In contrast, we find no relationship between either type of support and second conceptions. Because of the small increments considered (person/months), the coefficients related to conception propensities are all very low. They are even lower for the policy measures that operate on a scale of one percentage point increase in an average production worker’s wage.

**Table 1:** Selected results from fixed-effects linear probability models: The relationship between family policy orientations and first and second conceptions

	First conception		Second conception	
	Coeff	St.error	Coeff	St.error
Social invesment-oriented support	0.00003	0.00001	0.00001	0.00
Passive family support	− 0.00005	0.00000	0.00000	0.00
Rho	0.00089		0.00200	
Numaber of Countries	20		20	
Number of Observation	2,409,430		1,279,650	
AIC	− 5,560,143		− 2,573,641	
BIC	− 5,559,636		− 2,573,182	

Note: results are adjusted for educational level and year fixed effects in both models. In the first conception model, estimates are also adjusted for age and the non-proportionality of the educational level association over age groups. In the second conception model, estimates are also adjusted for age of the youngest child, age at first birth and the non-proportionality of the educational level association over age at first birth. *p < .05. **p < .01. ***p < .001

Supplementary Figure B shows the predictive margins of first conceptions according to the two policy orientations. If we consider the baseline (mean) conception probability to be 0.006 (x-axis at 0), investment-oriented support at its highest observed value (*x*-axis at 45 = 0.0074) is associated with a 22% higher first conception probability than at the mean, whereas traditional-oriented support is associated with 39% lower probability. At their lowest observed values, investment-oriented support is associated with a 27% lower first conception probability (*x*-axis at –55 = 0.0045) than at the mean, whereas traditional-oriented support is associated with 16% higher probability. In other words, an increase in social-investment support increases the probability of a first birth, whereas an increase in passive support lowers the probability of a first birth.

We next observe variation in the policy and fertility relationships through interactions. We interact one type of policy support at a time, while holding the other policy support constants. All interactions improve the model fit according to AIC/BIC statistics.

### First Child Conception Differences by Age and Educational Level

We first interact age with policy support. Full interaction results are displayed in Supplementary Tables D and E. How different types of policies are associated with childbearing of women at different ages is particularly relevant given the postponement of first births and increasing childlessness across Europe. Although we cannot distinguish whether postponement or childlessness underlies a negative relationship in these models, we would expect postponement to manifest as lower rates in younger ages (particularly when women are in their 20 s) and a recuperation at older ages (when women are in their 30 s). Childlessness is indicated by low first birth estimates for women in all older age groups.

**Fig 1:**
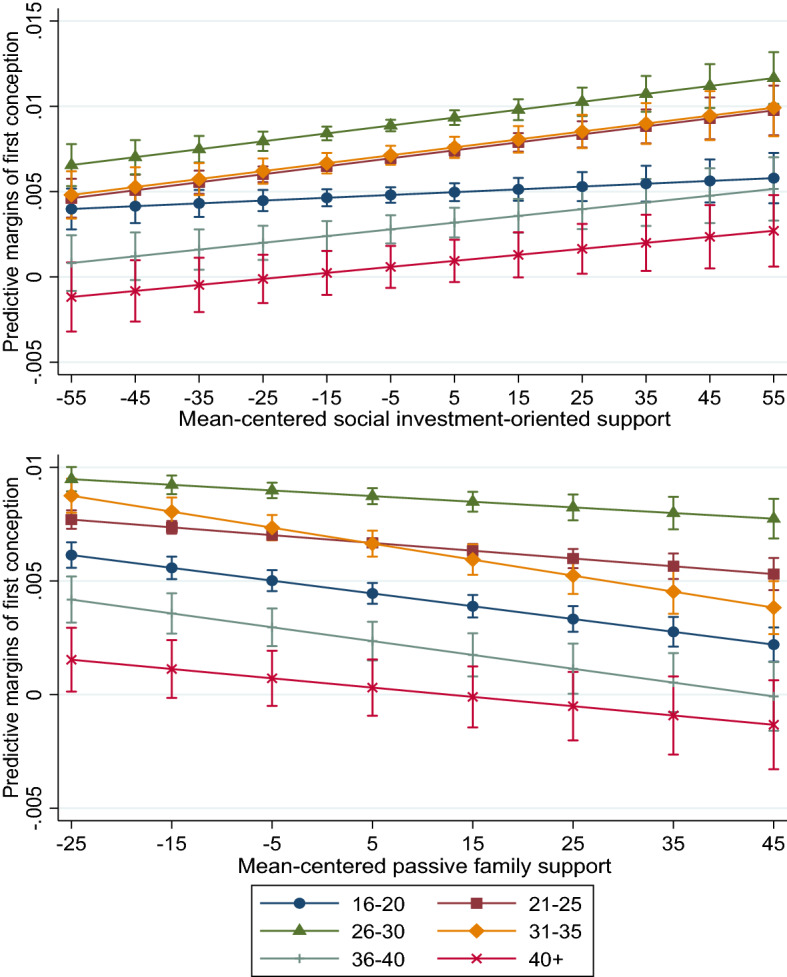
Predicted margins of first conception by an interaction of age and social investment-oriented support (top) and passive family support (bottom) results are adjusted for age, educational level, year fixed effects, the other form of policy support and the non-proportionality of the educational-level association over age groups; 95% confidence intervals in brackets

In Fig. 1, women aged 26–30 had the highest probabilities of entering parenthood followed by women on either side of that age range. As shown, higher social investment-oriented support is positively related to entering parenthood and this is evident across all age groups except for the very youngest (16–20). In contrast, all age groups show a lower probability of first conceptions as the level of passive family support increases. First, we note that the strongest negative trend appears for women who are 31–35. Given not having had a child up to that point, these women are the most likely to abstain if passive support increases. Women who were 16–20 and 36–40 had a similar negative reaction. In contrast, the effect for women in the 21–25 and 26–30 age group is only moderately negative. Note that the confidence intervals often overlap between points within the slope of a specific group, particularly when there are few women in that group, and overlap often appears at one end of the policy distribution but not the other.

**Fig 2:**
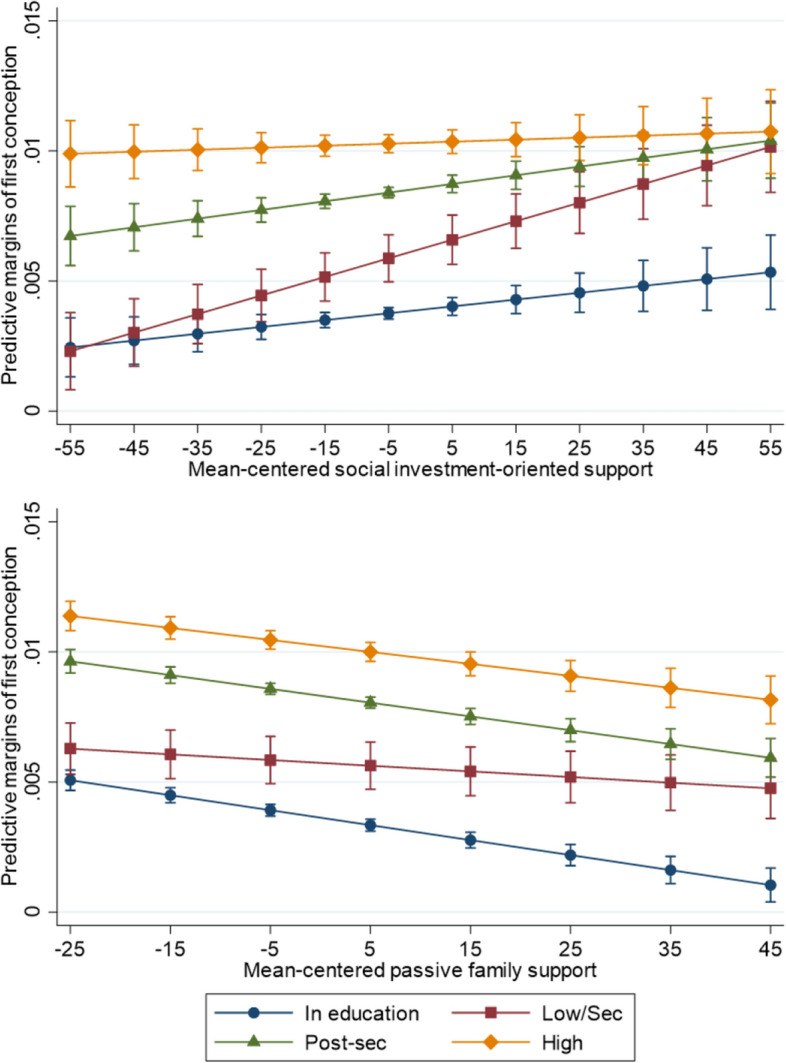
Predicted margins of first conception by an interaction of educational level and social investment-oriented support (top) and passive family support (bottom) results are adjusted for age, educational level, year fixed effects, the other form of policy support, and the non-proportionality of the educational-level association over age groups; 95% confidence intervals in brackets

Similarly, Fig. 2 provides estimates for an interaction between education and policy support. Full interaction results are displayed in Supplementary Tables F and G. The main effects in both figures show a positive gradient across educational levels, whereby the highest conception probabilities are among women who have achieved a high level of education and the lowest probabilities among women still in education. This corresponds to the common findings in which women who spend more years in education are more likely to quickly enter parenthood once finished with school.

We find that the lowest educated women are the most responsive to rising social investment support. The effect lessens at higher educational attainment, to the point that there is no relationship between social investment-oriented support and highly educated women’s childbearing. In contrast again, the increase in passive family support operates negatively for women who are studying and have high or post-secondary education. Women with low education, however, do not seem responsive to such changes in passive support.

### Second Child Conception Differences by Educational Level

**Fig 3:**
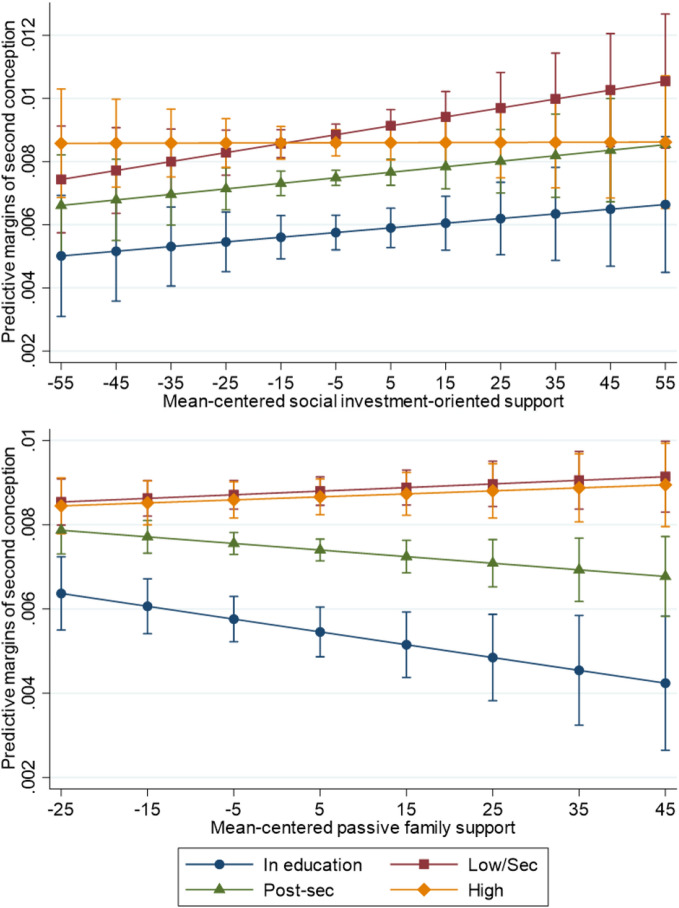
Predicted margins of second conception by an interaction of educational level and social investment-oriented support (top) and passive family support (bottom)results are adjusted for age at first birth, age of the youngest child, educational level, year fixed effects, the other form of policy support, and the non-proportionality of the educational-level association over age at first birth; 95% confidence intervals in brackets

Figure 3 displays the second conception probabilities as they vary across educational level. Full interaction results are displayed in Supplementary Tables H and I. The average effect of the policy measures was null in a model without interactions, but we see that this is clearly due to different associations across educational groups. As with the first birth, we find that the lowest-educated women are most responsive to increasing social-investment support, with post-secondary educated women and those in education still following only a slightly moderated positive trend of second conceptions. The highly educated are not affected by an expansion of investment-oriented support.

We find no differences between predicted estimates for the lowest and the highest educated women with rising passive family support. Women with post-secondary education and women who are studying, on the other hand, appear to be more likely to postpone or forego a second child when passive family support increases.

## Discussion

This is the first comparative study to analyse parity-specific fertility transitions at the micro-level alongside policy measures that change over time and reflect important differences in family policy orientation. It is also the first to apply the social investment approach of welfare state research as a theoretical framework in demography and to acknowledge the fundamental turn of welfare state orientation and social policies since the mid-1990s. This approach provides a framework that encompasses an employment as well as a gender-egalitarian perspective of fertility and thus unites economic as well as gender theoretical explanations of fertility behaviour. Despite much debate, neither the demographic propositions of the impact of different family-policy strategies on childbearing behaviour nor the core propositions of the social investment approach regarding its effect on social (in)equality have been tested on the micro-level before. There have also been no comparative studies that provide insight into how the ongoing and commonly incremental changes towards social-investment welfare states affect childbearing behaviour.

We focussed on the set of policies that are theoretically most contested, politically most varying and have given the most ambivalent results in macro-level analyses: family policies at the time of birth that either support women’s employment retention and facilitate gender-equal division of care or maintain a gendered pattern of care and work while reducing costs. These policies are the most relevant to the timing of family formation and of family expansion.

Which family policies increase fertility? Our results show that expanding social investment-oriented policies is related to higher first conception probabilities, whereas expanding passive family support leads to lower first conception probabilities. With their long-term employment perspective, social investment-oriented support was expected to provide incentives to establish oneself in the labour market before having a child, leading to postponement (lower first birth rates for young women). This incentive is further strengthened by the earnings-related parental leave predominant among social investment-oriented family policies. In fact, we do not find that younger women have lower first birth rates when social investment-oriented support increases. Instead, first birth rates increase similarly across all age groups except women aged 16–20. Because the age association is net of educational level and enrolment, we can assume that the unexpected finding for women who are in their early twenties relates to women who are in the labour force already. Women do work in this age group and many do not continue on to further studies. The expectation that women would be more likely to delay parenthood until they are established in the labour market may still be the case, and this seems to apply to women even in their early twenties. Overall, social investment-oriented support therefore appears to shape behaviour in a universal manner.

In contrast, passive family support is correlated with a lower probability of entering parenthood, and this appears to be the case across our age groups. This points to potential postponement and increased childlessness. Net of variation in social investment-oriented support, a high share of passive family support may be associated with a (long) care leave. Additionally, high passive family support that encourages women to remain at home to take care of a child may send a signal, particularly to employers, that women are not expected to maintain employment during the early child-rearing years. This may motivate women to postpone parenthood as late as possible to achieve a strong enough footing in the labour market and potentially accumulate savings, to allow them to take leave. It may also amplify the hesitation to have a child at all, which is revealed by the increasingly low probability that women will have a child under more passive family support if women have remained childless into their 30 s. Another explanation may be that in countries with very low family-policy support, e.g. the UK and the USA, the market has stepped in to provide childcare, whereas such options are largely lacking in countries with high passive family support (Meyers & Gornick, 2003).

Declining probabilities of having a first child are especially prevalent among highly and post-secondary educated women and those still in education. This is not surprising, since policies that focus on women as long-term carers may have an inhibiting impact on career options. Moreover, even high passive family support may not compensate for their future loss of income and employment trajectories (see also Aisenbrey et al., 2009). Social investment-oriented family policies were assumed to level out educational differences in employment opportunities (Korpi et al., 2013). which could also lead to similar childbearing behaviour among educational groups. Indeed, we found no differences between women with different educational levels with high social investment-oriented support. In fact, low-educated women were the most responsive to increasing social investment support, while more such support did not raise highly educated women’s first birth probabilities. Contrary to what one would infer from the Matthew effect arguments, more passive support did not increase first births among low-educated women. All of these results counter the theoretical claims of a general Matthew effect of social investment welfare states.

Our results also show that higher social investment-oriented support is positively correlated with second births, except among highly educated women, pointing to a stronger trend of family expansion when countries adopt social investment-oriented policies. That family expansion (second child conception) is positively linked to higher social investment-oriented support, while passive family support is not, corroborates past findings by Billingsley and Ferrarini (2014). This may again be due to the fact that passive family support may encourage women to reduce their labour force participation by taking long leaves (e.g. child care leave until the child’s third birthday).

Our study is limited by a few important shortcomings. We cannot control for urban/rural residence because we do not have migration histories to know where the respondents lived before the survey. Neither can we control for labour market participation nor partnership status, as information on work or partnership histories is not available for this large set of countries. In particular, knowing whether women were in paid employment during the window of time they were observed would have allowed us to observe the interaction between policies that are targeted at allowing the combination of work and family roles and those that are not. However, including this additional factor would not be correct to the extent that childbearing plans in the near future may condition women’s employment status in a given policy context. In addition, employment requirements for parental leave benefits vary across countries and time, and data on this variation would have been needed to calculate the eligibility of each woman. In our study, educational level is interpreted to indicate both opportunity costs of childbearing due to employment and attachment to the labour market.

As mentioned, no comparable data source exists on childcare coverage of 0–2-year olds, which we argue is the relevant time period for fertility decision-making and sets the stage for women’s employment patterns and gendered division of care in the home. Worth noting, however, coverage at this age range was low in most countries for the years that we covered. Although there is likely a correlation between high social investment-oriented support and high childcare coverage for 0–2-year olds as in the Nordic countries, Finland (as well as Norway during certain periods) also provided stay at home care-leave options. Thus, the correlation may not be as straightforward as one might suspect. Related to the family policies, our measures of policy support are based on a replacement rate of an average production worker wage. The benefits and limitations of this approach have been discussed in Ferrarini et al. (2013) and Wesolowski et al. (2020). Finally, interactions in fixed effects regressions may not provide true “within-unit” estimates when both variables that are in the interaction vary within units and one of them is correlated with an unobserved moderator of the other (Giesselmann & Schmidt-Catran, 2020). To the extent that this may be the case in our study, it is worth noting that interaction estimates remain generally consistent in terms of direction with the unbiased main effect of the two policy indicators and across all models.

Finally, we sought to estimate an average overall effect of policies across many countries that is net of country-specific differences in fertility. Policies may, however, have different effects in different countries, which large comparative research such as this cannot uncover. Further research seeking to understand how the relationship varies across countries would benefit from theorizing about important contextual differences.

In summary, we find that our results, covering 12 years of policy changes and fertility behaviour, provide a clear case for expanding social investment-oriented family policies as a means to avoid low fertility, whereas increasing passive family policies may not be a way out of low fertility levels. This holds for women of all educational levels. Although having the possibility to stay out of the labour market for a long time, as passive family policies offer, looks like a supportive family policy, women may increasingly judge this in light of their future employability and prioritize these future consequences. Such considerations may become even more prevalent in the future as more countries strengthen the social investment-oriented features of their labour market and social-security systems. By contrast, in countries that opt for social investment-oriented family policies—supporting women to stay in the labour market, maintaining the family’s financial basis, and promoting a gender-egalitarian division of work and care—women are less forced to choose between employment or having children. Women are therefore more likely to continue childbearing once they enter parenthood.

## Supplementary material

Supplementary file 1 (PDF 361 kb)

## Footnotes

1 There are numerous studies on the link between family policies and fertility, comparative and single-policy/country studies. Due to limitations in space, we mainly refer to studies that are the most relevant ones to our investigation.
