# Supplementary material for: Social Investment Policies and Childbearing Across 20 Countries: Longitudinal and Micro-Level Analyses
Source: Eur J Popul. 2022 Jun 30;38(5):951–74. doi: 10.1007/s10680-022-09626-3 (PMC9727052; doi:10.1007/s10680-022-09626-3)

Supplementary Table A. Descriptive statistics

	Parity 0 women		Parity 1 women	
Individual-level variables	% person-months	Person-months	% person-months	Person-months
Age				
16-20	39	931937		
21-25	31	731681		
26-30	16	377605		
31-35	8	189092		
36-40	5	108657		
40+	3	70458		
Education				
In education	43	1031622	10	124599
Low/secondary	19	450782	28	356539
Post-secondary	29	706636	45	577483
Higher	8	204003	17	211784
Unknown	1	16387	1	9245
Age of first child				
0-1			24	309749
2-4			23	294886
5-9			24	307915
10+			29	367100
Age at first birth				
16-19			17	222444
20-24			45	577437
25-29			25	324623
30-34			10	124627
35+			2	30519

Country-level variables	Mean	SD	Minimum	Maximum
Social investment-oriented support	-6.72	26.32	-41.89	60.89
Passive family support	-0.88	18.39	-27.89	49.12

Supplementary Table B. Results from fixed-effects linear probability model: First conceptions

	Coefficients		Standard errors
Education			
In education	-0.0081	***	0.0004
Low/secondary	-0.0058	***	0.0004
Post-secondary	-0.0026	***	0.0004
Higher	1		
Unknown	-0.0054	***	0.0012
Age			
16-20	-0.0007		0.0004
21-25	1		
26-30	-0.0029	***	0.0006
31-35	-0.0062	***	0.0006
36-40	-0.0101	***	0.0007
40+	-0.0115	***	0.0008
Education * age			
16-20 in education	-0.0016	**	0.0005
16-20 low/secondary	-0.0021		0.0013
16-20 post-secondary	-0.0012	*	0.0005
16-20 unknown	-0.0011		0.0017
26-30 in education	0.0057	***	0.0007
26-30 low/secondary	0.0061	***	0.0006
26-30 post-secondary	0.0044	***	0.0006
26-30 unknown	0.0045	**	0.0017
31-35 in education	0.0083	***	0.0009
31-35 low/secondary	0.0087	***	0.0007
31-35 post-secondary	0.0041	***	0.0007
34-35 unknown	0.0073	**	0.0021
36-40 in education	0.0084	***	0.0012
36-40 low/secondary	0.0076	***	0.0008
36-40 post-secondary	0.0030	***	0.0008
36-40 unknown	0.0091	**	0.0027
40+ in education	0.0081	***	0.0016
40+ low/secondary	0.0056	***	0.0009
40+ post-secondary	0.0020	*	0.0009
40+ unknown	0.0072	*	0.0031
**Country-level variables**			
Investment-oriented support	0.00003	*	0.00001
Passive family support	-0.00005	***	0.00000
Rho	0.00089		
Number of countries	20		
Number of observations	2,409,430		

Note: adjusted for year fixed effects, *p < .05. **p < .01. ***p < .001

Supplementary Table C. Results from fixed-effects linear probability model: First conceptions with interaction of age and investment-oriented support

		Coefficients		Standard errors
Education				
	In education	-0.0082	***	0.0004
	Low/secondary	-0.0057	***	0.0004
	Post-secondary	-0.0027	***	0.0004
	Higher	1		
	Unknown	-0.0054	***	0.0012
Age				
	16-20	-0.0009	*	0.0004
	21-25	1		
	26-30	-0.0029	***	0.0006
	31-35	-0.0063	***	0.0006
	36-40	-0.0102	***	0.0007
	40+	-0.0116	***	0.0008
**Country-level variables**				
Investment-oriented support		0.000047	***	0.000012
Passive family support		-0.000048	***	0.000006
Age * Investment-oriented support				
16-20		-0.000030	***	0.000004
26-30		0.000000		0.000006
31-35		0.000000		0.000007
36-40		-0.000007		0.000009
40+		-0.000012		0.000011
Rho		0.00095		
Number of countries		20		
Number of observations		2,409,430		
AIC		-5560194		
BIC		-5559623		

Supplementary Table D. Results from fixed-effects linear probability model: First conceptions with interaction of age and passive family support

		Coefficients		Standard errors
Education				
	In education	-0.0081	***	0.0004
	Low/secondary	-0.0058	***	0.0004
	Post-secondary	-0.0027	***	0.0004
	Higher	1		
	Unknown	-0.0053	***	0.0012
Age				
	16-20	-0.0007		0.0004
	21-25	1		
	26-30	-0.0029	***	0.0006
	31-35	-0.0063	***	0.0006
	36-40	-0.0101	***	0.0007
	40+	-0.0115	***	0.0008
**Country-level variables**				
Investment-oriented support		0.00003	**	0.00001
Passive family support		-0.00003	***	0.00001
Age * Passive family support				
16-20		-0.00002	***	0.00001
26-30		0.00001		0.00001
31-35		-0.00004	**	0.00001
36-40		-0.00003	*	0.00001
40+		-0.00001		0.00002
Rho		0.00091		
Number of countries		20		
Number of observations		2,409,430		
AIC		-5560165		
BIC		-5559593		

Supplementary Table E. Results from fixed-effects linear probability model: First conceptions with interaction of education and investment-oriented support

		Coefficients		Standard errors
Education				
	In education	-0.0079	***	0.0004
	Low/secondary	-0.0051	***	0.0004
	Post-secondary	-0.0024	***	0.0004
	Higher	1		
	Unknown	-0.0053	***	0.0012
Age				
	16-20	-0.0006		0.0004
	21-25	1		
	26-30	-0.0029	***	0.0006
	31-35	-0.0062	***	0.0006
	36-40	-0.0099	***	0.0007
	40+	-0.0112	***	0.0008
**Country-level variables**				
Investment-oriented support		0.000001	*	0.000013
Passive family support		-0.000047	***	0.000006
Education * Investment-oriented support				
In education		0.000019	**	0.000001
Low/secondary		0.000064	***	0.000008
Post-secondary		0.000026	***	0.000007
Unknown		0.000003		0.000026
Rho		0.00090		
Number of countries		20		
Number of observations		2,409,430		
AIC		-5560234		
BIC		-5559676		

Note: adjusted for year fixed effects and an interaction of education and age, *p < .05. **p < .01. ***p < .001

Supplementary Table F. Results from fixed-effects linear probability model: First conceptions with interaction of education and passive family support

		Coefficients		Standard errors
Education				
	In education	-0.0081	***	0.0004
	Low/secondary	-0.0058	***	0.0004
	Post-secondary	-0.0026	***	0.0004
	Higher	1		
	Unknown	-0.0064	***	0.0013
Age				
	16-20	-0.0007		0.0004
	21-25	1		
	26-30	-0.0029	***	0.0006
	31-35	-0.0062	***	0.0006
	36-40	-0.0101	***	0.0007
	40+	-0.0115	***	0.0008
**Country-level variables**				
Investment-oriented support		0.00003	**	0.00001
Passive family support		-0.00005	***	0.00001
Education * Passive family support				
In education		-0.00001		0.00001
Low/secondary		0.00002	*	0.00001
Post-secondary		-0.00001		0.00001
Unknown		-0.00008		0.00005
Rho		0.00092		
Number of countries		20		
Number of observations		2,409,430		
AIC		-5560165		
BIC		-5559606		

Note: adjusted for year fixed effects and an interaction of education and age, *p < .05. **p < .01.

***p < .001

Supplementary Table G. Results from fixed-effects linear probability model: Second conceptions

	Coefficients		Standard errors
Education			
In education	-0.0057	***	0.0006
Low/secondary	-0.0018	**	0.0006
Post-secondary	-0.0027	***	0.0005
Higher	1		
Unknown	-0.0004		0.0025
Age at first birth			
16-19	1		
20-24	-0.0020	***	0.0005
25-29	-0.0051	***	0.0006
30-34	-0.0083	***	0.0008
35+	-0.0131	***	0.0014
Age of youngest child			
0-1	1		
2-4	0.0015	***	0.0002
5-9	-0.0057	***	0.0002
10+	-0.0105	***	0.0002
Education * age at first birth			
20-24 in education	0.0015	*	0.0008
20-24 low/secondary	0.0002		0.0007
20-24 post-secondary	0.0010		0.0006
20-24 unknown	-0.0053		0.0029
25-29 in education	0.0052	***	0.0009
25-29 low/secondary	0.0044	***	0.0008
25-29 post-secondary	0.0028	***	0.0007
25-29 unknown	-0.0019		0.0030
30-34 in education	0.0069	***	0.0014
30-34 low/secondary	0.0058	***	0.0011
30-34 post-secondary	0.0035	***	0.0010
30-34 unknown	0.0043		0.0035
35+ in education	0.0041		0.0027
35+ low/secondary	0.0053	**	0.0017
35+ post-secondary	0.0033		0.0017
35+ unknown	-0.0003		0.0051
**Country-level variables**			
Investment-oriented support	0.00001		0.00001
Passive family support	0.00000		0.00001
Rho	0.00200		
Number of countries	20		
Number of observations	1,279,650		

Note: adjusted for year fixed effects, *p < .05. **p < .01. ***p < .001

Supplementary Table H. Results from fixed-effects linear probability model: Second conceptions with interaction of education and investment-oriented support

	Coefficients		Standard errors
Education			
In education	-0.0055	***	0.0006
Low/secondary	-0.0014	*	0.0006
Post-secondary	-0.0025	***	0.0005
Higher	1		
Unknown	-0.0003		0.0025
**Country-level variables**			
Investment-oriented support	0.0000004	*	0.000017
Passive family support	0.000002		0.000008
Education * Investment-oriented support			
In education	0.000014		0.000012
Low/secondary	0.000028	**	0.000010
Post-secondary	0.000017	*	0.000009
Unknown	0.000012		0.000043
Rho	0.00198		
Number of countries	20		
Number of observations	1,279,650		
AIC	-2573641		
BIC	-2573134		

Note: adjusted for year fixed effects, age at first birth, age of youngest child, and an interaction of age at first birth and educational attainment, *p < .05. **p < .01. ***p < .001

Supplementary Table I. Results from fixed-effects linear probability model: Second conceptions with interaction of education and passive family support

	Coefficients		Standard errors
Education			
In education	-0.0058	***	0.0006
Low/secondary	-0.0017	**	0.0006
Post-secondary	-0.0027	***	0.0005
Higher	1		
Unknown	-0.0011		0.0027
**Country-level variables**			
Investment-oriented support	0.000016		0.000016
Passive family support	0.000007		0.000010
Education * Passive family support			
In education	-0.000038	*	0.000016
Low/secondary	0.000001		0.000011
Post-secondary	-0.000023	*	0.000011
Unknown	-0.000062		0.000070
Rho	0.00191		
Number of countries	20		
Number of observations	1,279,650		
AIC	-2573644		
BIC	-2573138		

Note: adjusted for year fixed effects, age at first birth, age of youngest child, and an interaction of age at first birth and educational attainment, *p < .05. **p < .01. ***p < .001

Supplementary Figure A. Investment oriented-support and passive family support over time in 20 countries: Percent of an average production workers' wage

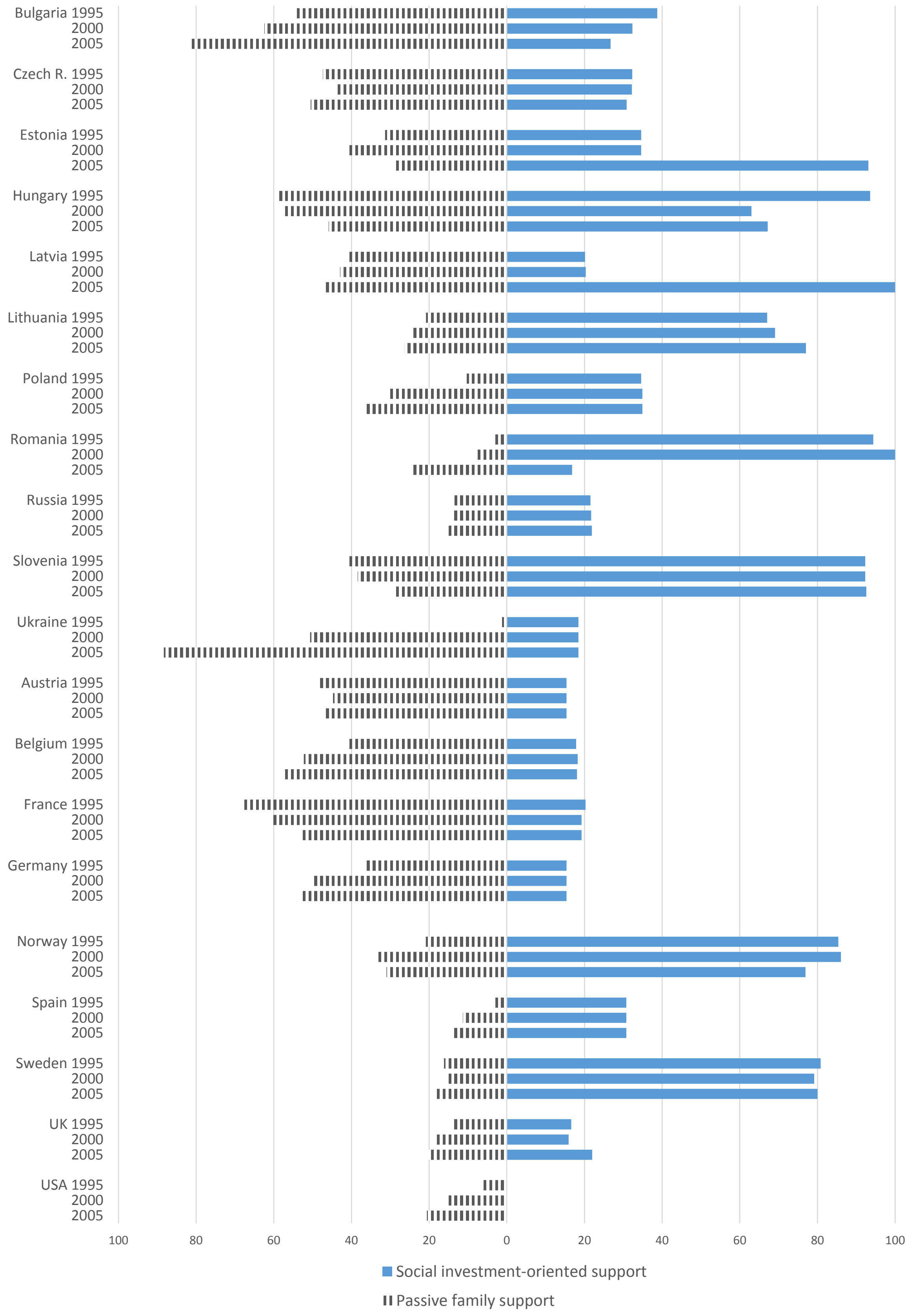

Supplementary Figure B. Predictive margins of first child conception according to policy support

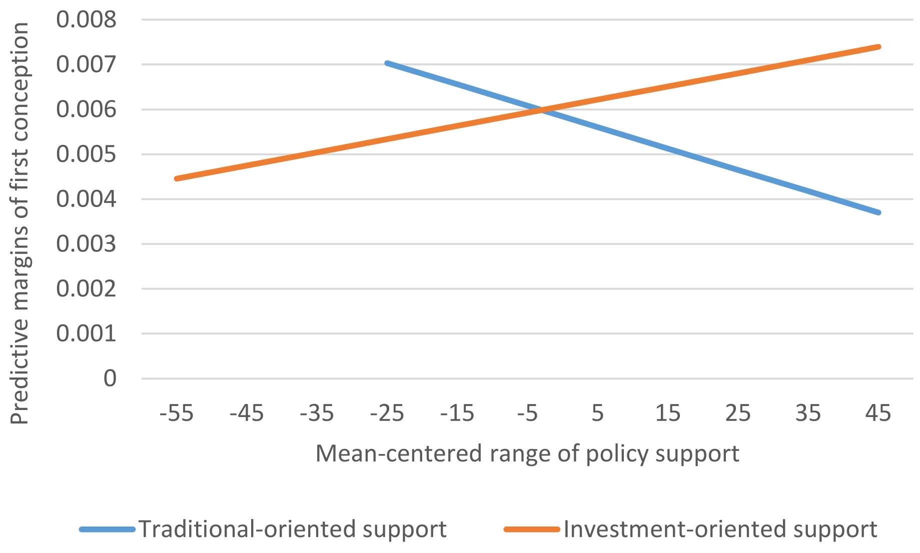

Supplement: Supplementary file 1 — (PDF 361 kb) [file 10680_2022_9626_MOESM1_ESM.pdf]
